# Training in Open Dialogue and Dialogical Practice: creatively responding as trainers and writers

**DOI:** 10.3389/fpsyg.2023.1174680

**Published:** 2023-10-04

**Authors:** Catherine Thorley, Judith M. Brown, Mia Kurtti, Alita Taylor

**Affiliations:** ^1^Independent Practitioner, Essex, United Kingdom; ^2^School of Social Sciences, University of NSW, Sydney, NSW, Australia; ^3^Private Practice, Sydney, NSW, Australia; ^4^Faculty of Social Sciences, University of Helsinki, Helsinki, Finland; ^5^Lapland Wellbeing Service County, Kemi, Finland; ^6^Open Dialogue Pacific, Lynnwood, WA, United States

**Keywords:** Open Dialogue, training, dialogical practice, dialogical process, reflective supervision, embodiment, open dialogue training, dialogical training

## Abstract

This paper emerges from a series of conversations about training in Open Dialogue and dialogical practice. In our dialogue, we found ourselves moving away from seeking definitive answers about content (what to include) or process (how to include). We asked, “Why are we asking this question about training at all?” Maybe it is because many helpers and all kinds of professionals all over the world are truly asking, “How do we do, or how do we learn how to do ‘open dialogue’?.” That question starts with “How to train others in the practice?”

We moved toward responding to our own questions—what are we offering as trainers and what are the trainees seeking? We sought to explore what is required for a training space that accommodates the hopes of both trainers and trainees. Words arose during our talking, and we listened to them, let them sink in, and reflected on them. Some words resonated with us as trainers; some linked with observing trainees’ experiences (including our own); some showed a glimpse of the relationship between trainer and trainees. These emergent words point to a series of learnings, aspects of the training that we as trainers have come to believe are important. The following paper expands upon these words while also including actual portions of our dialogues and vignettes from training. As such, we illustrate our ongoing learning as trainers of Open Dialogue and dialogical practice as it occurs within the unique nature of each training we provide.

## Introduction

Hello and welcome to our article, which will explore our experiences and discussions about being trainers in Open Dialogue and dialogical practice in our four different countries and contexts. What is the history of how we came to be writing this article? At the start of the pandemic, a group of women came together to support one another as writers. We met online monthly, with our first challenge being to find times when meeting from our different time zones in Finland, the United Kingdom, the United States, and Australia would be possible. With some compromise, early morning for some and late evening for others, we managed it. Through the months, our connection deepened as we came to our meetings with no agenda. We just listened and were heard. To see and to be seen kept us returning. From this space, ideas emerged. Over time, we had used the space to talk about our different training experiences, so when the invitation to write for this journal arrived, we were all keen to write together. In the spirit of dialogical practice, we want to be transparent about what we are inviting you into. In essence, we are exploring the qualities and processes that are part of dialogical practice and training. Primarily, our focus is on how to “be”, less so on how to “do”.

Open Dialogue as an approach is a model and frame for organizing mental health services. There is a global shift in thinking, in which people are exploring the contribution of Open Dialogue and dialogical practice to mental health services. Questions around training are present, and different training programs are being developed. As increasing numbers of people are thinking about these issues, it makes sense that there are still many open questions about skills and insights regarding the Open Dialogue system of care and dialogical practice. These questions arise when designing an adequate training program.

In writing this paper, we offer our contribution to this conversation. Open Dialogue is a paradigm shift in mental health, from an expert and set agenda about symptom reduction, to a dialogical focus on relationships, understandings, and stories. Our writing reflects this shift. As such, our process here mirrors a dialogical way of working, whether as a therapist in network meetings, a supervisor, or a trainer. Although we could describe this work as part (poly)-auto-ethnography and part perspective, the dialogical nature of its methodology may suggest that this work is unfinished, akin to the unfinalizability of network meetings. In taking a ‘not knowing’ approach (Anderson and Goolishian, 1992) to stay curious, we privileged listening and responding over the certainty of theory or a predetermined destination. As with network meetings, we aimed to be in the present moment with the writing as it unfolded, to listen and to respond to what emerged from each of us, to privilege our relationship with each other, and to bring more of ourselves to the writing. We sought to stay with our differing voices, feelings, and emotions and held space for silences between and within our meetings. We sought to honor the emergence of the many voices within each of us, as with network meetings. In the writing, we trusted in the dialogue and in the dialogical process.

We invite you into this dialogue, into trusting in the dialogical process, where perhaps there are times when there is no clear concept of where it is going. Just like at the start of any dialogical training session or network meeting we ask, “What would you like to talk about today?” As with dialogical practice and training, we are not aiming for a set response from you but for as many different responses as there are readers. To quote from the chapter named, “Creativity in the whole life” in a special section, “Dialogue and Culture”, “...it must be stressed that what follows is not given in the spirit of a prescription that society follow. Rather it is an invitation to the reader to begin to investigate and explore in the spirit of free play of ideas and without the restriction of the absolute necessity of any final goal or aim” (Bohm and Peat, 1987). Perhaps it would be helpful to take a moment and consider how you are entering this space. What might be your curiosities and wonderings? What are your feelings? Is there any sensation in your body at the start of this? As you read on, perhaps you will notice how your thoughts, feelings, and bodily sensations move and change in the process.

As you read, you may also notice that some words are in italics. These are words that have stood out for us. They seem to be the qualities that we seek to engender during the differing forms of training that we each offer. Maybe they are also the challenges of training in a dialogical way. So now, we share our reflections with you in this space between our words and your experiences.

### Mia’s Voice

When I began to attune to this topic, the very first thought for me was the beginning. How do we start the training process? What is there for all of us to think about in terms of creating a fully competent Open Dialogue and Dialogical Practice training program that would *offer* people a suitable framework for learning?

It is unbelievable how the core *presence* of people is the same all over the world: People want to connect and create dialogical spaces. This helps! (Mia’s trainer’s notebook, 2017)

People require dialogic skills in their practice when meeting people and networks in distress. How do we design training that supports the learning process which offers trainees the possibility of making the required changes in their practice? How do we invite the trainers and the trainees to a joint journey where knowledge is generated so that trainees may become dialogic practitioners—practitioners who also have the required insight into different levels of Open Dialogue as a practice and/or system of care? There are many questions for trainers to discuss together when planning the process collaboratively. When creating the general frames, it is also important to embrace that every learning process is unique and that *each person needs time and space to find their own way and at their own pace*.

In my experience, the planning of the training program requires consideration of the context of the training. We should *honor the local* prospects in building the frames and circumstances that enable people to have an empowered position in relation to the new approach they are learning about. What are the needs for dialogical training in different organizations around the world? How can we build the *training in a need-adapted manner while respecting the core principles* of an Open Dialogue practice (Seikkula and Alakare, 2004)? When we establish a training program, what are we *inviting* people into? I am wondering if the trainees have enough information about the purpose of training in their context so that they can bring their own needs accordingly. Trainees could be encouraged to ask themselves, ‘Are my needs met here and how do I bring my questions to the process?’

I am also carrying my own history and context as a practitioner and trainer coming from Western Lapland, Finland. Open Dialogue and dialogical practice in Western Lapland could not have taken place without extensive training over several years. The practice has been supported by dialogic family and network-based therapy training that has been offered to all the workers in the department of psychiatry and the larger community. Learning through intertwined aspects of theory, supervision, and family of origin processes, people are invited to create a dialogical dimension to their practice. The work has shown that dialogue in network meetings requires *trust* between team members and also a sound understanding of the use of reflective practice to generate insight into the topic and situation at hand. Practitioners also need to be able to *listen* to both outer and inner dialogues when facilitating the meetings. Each participant in the meeting *reflects* the voices of multiple roles, identities, and experiences carried and held within a narrative and a bodily memory (Haarakangas, 1997; Haarakangas et al., 2006). A multifaceted dialogue can arise from these aspects, which can offer crucial new and different insights for participants when the practitioner–trainer has an awareness of their own inner voices, including how these voices emerge in their professional role at any moment.

One perspective that I feel is crucial is that trainers need to have the experience of being with people in mental distress and *bodily knowing* about the nature of processes (Lyons-Ruth, 1998; Shotter, 2011). This can help them generate self-agency in the trainee’s learning process (Rautkallio, 2019). The main goal for me is that in the end, it is the process, and the trainees in it who have been making the process, and trainers have the privilege to witness (i.e., “with”-ness) it.

### Alita’s Voice

What is open dialogue training? Sometimes, I wonder, is “training” the right word? What do others think when they hear the words “open dialogue”? Is it a “thing” they hope to “implement” or to change others with somehow? The ways of learning information in chunks and bits of formulaic knowledge, historically fed to us by the powers that be, are changing. Embodied, implicit knowing, or dialogical knowing is a bit different (Lyons-Ruth, 1998; Brown, 2015). If or when a facilitator, trainer, or teacher can engender a space where trainees are invited to move more toward that embodied, implicit, or dialogical moment and maybe a bit away from the formulaic, perhaps dialogism begins. What I mean to say is that there are bodies in a room together, whether in a treatment meeting or a training/learning environment, and these bodies come with implicit knowledge. How do they know what they know? And can we as trainers per se ask ourselves whether we are acting on or initiating movement toward or away from the co-knowing that might be coming from the “meeting” of the other bodies in the room? There are these moving-toward and moving-away movements happening all along. Are we attending to these?

On its way to the ocean, is the river’s edge where the bravest of settlers surrenders into the way.

What is going on in our waters? What is happening in our world? What can be learned of it in partnership, in collaboration that can never be learned in hierarchy …

Pause

(On Its Way … by Alita Taylor)

One thing that keeps pressing upon me at different times while pondering questions about dialogical training spaces is “Who trains?” What about the trainer? Are they humble, open, and trustworthy? Do they actually care about their trainees? How do they feel right now, right here in the moment with me? Will they be honest? Are they wanting to help me “get” something that they wish I would “get” but have not yet? Who are the ones who can teach us something? What are the ways in which we are teachable, and what are the ways that teachers or trainers themselves stay present, compassionate, and loving? Are they hungry? And are they open to all the ingredients here in the room to make a sort of soup of learning together?

How do we train? Taking together our own preparedness of material (e.g., psychodynamic and systemically-based exercises, family-of-origin and supervision homework brought to life in a reflective process facilitated by the trainer, impromptu role-plays), we cannot forget to ask trainees throughout training days, “How would you like to use this space today?” We negotiate together about how to use the space and trainers co-construct a safe space. But what is a safe dialogical space (Simon, 2023)? We cannot know this without curiosity and asking and listening to those in the room. The question is: What happens to the space when it is not negotiated beforehand or when one or more voices are more powerful? What is that like for the trainers and the trainees? What is coming up for the trainees? Making enough space available for exploring and struggling together. Trainers participate as *containers*, *holding space*, having the willingness to share the space, practicing co-regulation. We check in together. “How is this for you in this present moment?” Wondering together is a creative process in the here and now within any given moment in any given role-play or other reflective training exercise together.

Dialogism is like being with a child. Waiting and stopping, not saying everything on our mind. Allowing time for digesting, listening to the meaning-making, like making a recipe with your hands, not with imitation butter flavor, but being in the flow, wondering, asking without expectation of a certain answer, without a “right way”, trusting the agency of the organizing happening in the here and now. Trusting in the agency of the organism, in all the beings in the here and now in this new meeting or training in which all are participating, of which all matter. How to “elegantly order” the voices, the bodies housing the voices, the helpers near and far, engaging, and realizing the contexts? How uncertainty can be a bridge, a common grief expressed, a holding environment, like the improvisation of a dog playing in the water.

We aim to be *invitational* in the exercises we offer, and we try to remember our power as trainers and how hard it might be to decline invitations. The depth and topic of sharing are up to the trainee in any given exercise. We also aim to give space and time for everyone to *reflect on bodily, emotional, and cognitive responses* and to process these as individuals and within groups. One such important training practice is called the Wheel of Awareness (Siegel, 2018), in which individuals and groups can practice all the different ways one can be aware. These positionalities can be developed and can bring the right hemisphere ways of knowing to the fore. *Giving space and time* to the process of what is to be learned together is imperative. It can not be and is not the same every time. There are always new moments and new thoughts to be shared and responded to Cunliffe and Lock, 2020. The “how” we train is inside us. It is in how we see and respond to what is happening and in how we collaborate with others in that space to talk (Anderson, 2014). Wondering together and leaving room. Being willing and able to let go of fixed positions, opening to the free play of thought in a spirit of goodwill and friendship, ready to acknowledge any fact and any point of view as it actually is—this generates a dialogical culture in training spaces (Bohm and Peat, 1987).

### Cathy’s Voice

I am on the telephone, listening, and talking with a person (who we might call the person who accesses services) and another person (who we might call a colleague). That said, by being dialogical, these positions do not feel so rigidly defined. We share what is on our minds, and what we are sitting with, and sometimes sit in silence as thoughts and feelings and bodily sensations arise in the space. I am moved by what is shared and by what I learn about myself. I feel my thoughts and ideas expanding. The “person who accesses services” says that we should call this way of working, “loving and nurturing”.

I am now in a new country, having arrived at 2:00 a.m. It is incredibly hot. Walking to the venue where we will be training, I have tears in my eyes. *We bring all of ourselves* to the training. My heart is full. My colleague begins the training day by talking about her mixed connections to the country. She has brought herself to the space and her own different inner and outer voices. She shares something of her own vulnerability. A safe space feels like it is opening whilst keeping aware of the transient and complex positions of safety in group spaces. In the moment, the trainees experience this and begin to respond with their own feelings and thoughts. In the afternoon, we meet for lunch with a family who we have previously only met online. Meeting them in person, I feel a rush of joy and connection. Love. I am buying drinks, and one of the family members is helping me. The father says, “Your brother is helping you to buy tea.” This resonates with us all.

The family has agreed to join the training. They sit in an inner circle with me and another Open Dialogue trainer. We have both facilitated previous network meetings with the family online. The trainees sit in an outer circle around us. We had previously agreed with the family to all speak about the family’s experience of the meetings rather than having a live network meeting. Today, the family is discussing having a network meeting. I am more hesitant, since the family has only just met the training group and am *transparent* about this and we enter *not knowing*. The family members begin by talking about their experience of the meetings, how they work and how they feel.

“We are just asked what we want to talk about.”

“The practitioners speak about what they are feeling together so that we can hear it.”

“It is like sitting with family.”

“We don’t feel judged.”

“We all feel listened to.”

Then one of the members of the family begins to speak about something he had done that he was troubled by. It seems that a safe enough space has been created for him to bring his own vulnerability here and feel brave enough speak of it. Those of us in the inner and outer circles lean in attentively as the family speaks together about this. The pace is slow, and there are a lot of silences. Tears are shed from people in the inner and outer circles. It is hard to put into words but it feels like time has slowed down and that there is a feeling of connection and love in the space between us all.

After the meeting, the family and the trainers who have met with the family go to a local cafe together whilst the other trainers and trainees pick postcards with different images to write a few words to the family about what has moved them. Before leaving, the family speaks informally to the other trainers and trainees. More heartfelt connections and sharing take place. One of the trainees stands as the family exits as a mark of gratitude to the family.

We had all taken the journey together, with *both the trainers and trainees contributing to creating an embodied sense of safety and love in the room* (Seikkula and Trimble, 2005). In addition to direct teaching on *how* to coordinate a network meeting, the trainees expressed that they felt an understanding of what it was like to be part of one. The next day, my colleague and I spoke about our experience of the meeting in a reflective supervision space. We sometimes call this “intervision” because of its *flatter hierarchy*. We invited three trainees to listen and to be part of a reflecting team. We spoke, not about the content of the meeting, but about ourselves and what came up for us. More tears were shed. In the spirit of dialogical practice, the reflecting team in their supervision space spoke of their connections to our words and did not interpret, offer solutions, or advice. One trainee said that she felt envious that we had a space like this where we could *trust one another enough to share our vulnerabilities.* Another trainee said that they now knew how dialogical practice felt, adding that nothing had really changed in their understanding, but that something had shifted for them.

### Judith’s Voice

Years ago I bought a card from one of my special places in the world, with the words ‘To discover the ocean, one must first lose sight of the shore’. Indeed. My exploration of dialogical practice has led me into learning and training and learning and…and so it goes on, continuing to beckon me...into that ocean. The early mixture of both fear and excitement has calmed over time, yet the dialogical process continues to surprise me….

We are on the third day of a 4-day introductory training in Open Dialogue and dialogical practice. Most trainees in the group have ostensibly settled into the shift away from didactic training, toward a dialogical training experience with *a focus on both content and process*. Since the first day, I have been aware of an older man sitting in the circle of chairs, at ten o’clock to my six o’clock position in the circle. I have a sense that he is less engaged with the training, less engaged with the group, and less engaged with me. I have wondered if perhaps he has been told to attend, or perhaps it is related to my gender, age, or professional discipline. When he directs questions at me, I metaphorically and actually lean into the space between him and I, *to stay with*—yet not be overcome by—his presence.

And on the third day, a question emerges, not from the older man I had been aware of, but from another in the circle. “But you need to tell us how to do Open Dialogue.” Once it is voiced, everyone seems to breathe out. I encourage the trainees to remain in the unknowing and lack of definition for now. I *encourage* them to trust that a training process that remains congruent with a dialogical way of working—a dialogical way of being—will bear fruit. This response seems to settle the group. Or perhaps the voicing of the question has already done so.

On the following day, the last of the training, there is a calmness in the space...as usual. Everyone seems settled, including the older man. He approaches me before leaving. Something in him—and in me too—has shifted. It is *a sense, an inner knowing*. It is as if we have come full circle now, together. I have *hope and trust in the dialogical process* of training yet again, but what happens is still a surprise.

These moments bring to mind the need to touch on the nuanced experiences of previous training—to trust in the dialogical process. Whether in training, supervision, or clinical work, it has taught me to trust that each person in the room is experiencing their own process, while also experiencing a group process. As a dialogical practitioner, trainer, or supervisor, the *dialogical process in the group is for me to manage, but the process for each individual is theirs alone.* This is most apparent in the refrain that has emerged in every 4-day Introduction to Open Dialogue and dialogical practice training. It is the moment of the question asked by trainees: “Tell us what to do. How do we do Open Dialogue?”

In past trainings, this question often remains unspoken until the third day, to be released, or perhaps it escapes with the momentum of sitting for days with uncertainty. Often, it emerges from one trainee, before it echoes elsewhere around the circle. In dialogical training, such a question opens the possibility for trainees to experience trusting in the ever-emerging dialogical processes within the group, and within themselves. It seeks a trainer’s response of holding steady, *showing rather than telling*. For both trainees and trainers, the question invites everyone to *wait for the learning to emerge*, as befits their way of learning, their way of being. The question is part of the training process. As is the response.

In trusting in the dialogical process of training, a dialogical space is created (one could say composed) by, and in the group. In this space, dialogical concepts can be introduced, begin to be processed, and possibly start to be integrated into each individual’s practice and way of being. To differing degrees, it is different for everyone. Each trainee gradually learns to be dialogical, in individual and group processes. So too, it is for the trainer. Becoming dialogical is unfinalizable, it is never complete (Bakhtin, 1984).

## Our Conclusion Is to Pause

Thus, in coming together in a dialogical way, we do not conclude here but come to a pause. We wonder, “Have we used the space to talk about what you wanted to talk about today?” Our writing has been something of a dialogical journey for us, with the ideas and words unfolding as we continued to talk, to listen, to reflect, and to trust that something would emerge. Now we invite you to consider yourself in this dialogical process. What is coming up for you? What are you noticing about yourself? We would like to invite you to take a moment to notice these things … in this moment.

(On Its Way continued … by Alita Taylor)

On its way to the ocean, is the river’s edge where the bravest of settlers surrenders into the way.

What is going on in our waters? What is happening in our world? What can be learned of it in partnership, in collaboration that can never be learned in hierarchy.

Miracles I see in our hands, and with the ones next to us.

Bluer than the sea are our woven sorrows of which we must hold with exquisite care.

Who are the helpers like this? Where are the servants like this, who care enough to reveal the sadness that our psychological, behavioral health interventions have lost their way, who say strongly like the river does—we know not where we are going. We know not tomorrow’s weather, but we know we must flow with what is, and fight not the rocks of time. Instead, we collect light, fall free, making whirlpools of wonder, leaving nature’s job to all the elements.

We are not visitors, nor individual inventors. We are together in this, beyond science and categories.

We are artists walking around with instruments called bodies with voices, cries, aches and ideas. We shall experience it all with one another because when there is no one there to hear and see another’s experiences our Body loses life limb by limb.

At the start we revealed that particular words/concepts italicized throughout were the things which emerged for us as qualities we seek to engender in a dialogical training. The qualities refer to the *being with* throughout training in Open Dialogue and dialogical practice whether in the dialogical family-of-origin/social network exercises, supervision, or theory days of training. They include our desire or intent to honor the local, to be invitational in our offerings, to reflect on bodily, emotional, and cognitive responses, to listen for and remember the not-knowing-ness, to be transparent, to hold dialogical space, to be a container, to give the time and space for each to find their way in their own pace, to attend to the bodily knowing happening, to wait for learning to emerge, to trust one another enough to share our vulnerabilities, both trainers and trainees contributing to creating an embodied sense of safety and love in the room, a flatter hierarchy, hope and trust in the dialogical process, staying-with, bringing all of ourselves, showing rather than telling, encouraging and sensing-into our inner knowing, focusing on both content and process, reflecting and adapting to the ever-changing needs of both trainees and ourselves as trainers seeking to provide the conditions for dialogue.

These are the conditions that we as four women from four different parts of the world seek to provide as we continue to meet online together to support one another’s work and lives. During the pandemic, we needed each other, and we continue to do so. We feel the importance of continuing dialogue with other trainers in a safe dialogical space is a necessary part of being dialogue facilitators. We, too, involve ourselves in a process, making ourselves vulnerable, sharing with one another our internal dialogue, our worries and hopes, and the difficulties and the joys of walking the path of a trainer in Open Dialogue and dialogical practice. We alone cannot know what to include, what to exclude, or what curriculum should evolve, or how, but we continue to share our experiences and to be open and creatively responsive to what is needed in the training we offer. What contexts are we bringing ourselves into? Who holds power? Where is our own power in what we are making space for? By continuing to support and hear the struggles and wonders of training experiences, we continue to learn more. We remain in an ongoing process.

## Data availability statement

The original contributions presented in the study are included in the article/supplementary material, further inquiries can be directed to the corresponding author.

## Author contributions

All authors listed have made a substantial, direct, and intellectual contribution to the work and approved it for publication.

## Conflict of interest

The authors declare that the research was conducted in the absence of any commercial or financial relationships that could be construed as a potential conflict of interest.

## Publisher’s note

All claims expressed in this article are solely those of the authors and do not necessarily represent those of their affiliated organizations, or those of the publisher, the editors and the reviewers. Any product that may be evaluated in this article, or claim that may be made by its manufacturer, is not guaranteed or endorsed by the publisher.
